# Prioritization of anticancer drugs against a cancer using genomic features of cancer cells: A step towards personalized medicine

**DOI:** 10.1038/srep23857

**Published:** 2016-03-31

**Authors:** Sudheer Gupta, Kumardeep Chaudhary, Rahul Kumar, Ankur Gautam, Jagpreet Singh Nanda, Sandeep Kumar Dhanda, Samir Kumar Brahmachari, Gajendra P. S. Raghava

**Affiliations:** 1 Bioinformatics Centre, CSIR-Institute of Microbial Technology, Sector 39A, Chandigarh, India; 2 CSIR-Institute of Genomics and Integrative Biology, Mathura Road, New Delhi-110007, India.

## Abstract

In this study, we investigated drug profile of 24 anticancer drugs tested against a large number of cell lines in order to understand the relation between drug resistance and altered genomic features of a cancer cell line. We detected frequent mutations, high expression and high copy number variations of certain genes in both drug resistant cell lines and sensitive cell lines. It was observed that a few drugs, like Panobinostat, are effective against almost all types of cell lines, whereas certain drugs are effective against only a limited type of cell lines. Tissue-specific preference of drugs was also seen where a drug is more effective against cell lines belonging to a specific tissue. Genomic features based models have been developed for each anticancer drug and achieved average correlation between predicted and actual growth inhibition of cell lines in the range of 0.43 to 0.78. We hope, our study will throw light in the field of personalized medicine, particularly in designing patient-specific anticancer drugs. In order to serve the scientific community, a webserver, CancerDP, has been developed for predicting priority/potency of an anticancer drug against a cancer cell line using its genomic features (http://crdd.osdd.net/raghava/cancerdp/).

Due to advancements in the field of sequencing technology, whole genome of different types of tumor cells have been sequenced. This flood of genomic information of tumors has broadened our understanding and provided valuable insights related to molecular and genetic characteristics of cancer types^1^,^2^. These sequencing efforts have now forced the scientists to change their view to accept that each individual tumor has its own genetic characteristics and is different from the other tumor even if they both belongs to the same tissue^3^. This is the reason that patients having similar cancer responded differently to identical chemotherapeutic drugs. Therefore, it is highly recommended to treat individual tumor as a different disease to make the treatment more effective with lesser side effects. This is the reason that researchers are focusing on personalized medicine or patient/tumor-specific drugs where aim is to identify right drug to right person at right time^4^,^5^.

In the recent past, few large-scale pharmacogenomics studies, namely the cancer genome project (CGP)^6^, and cancer cell line encyclopedia (CCLE)^7^ have been published. Both studies provide genomics data of large panel of cancer cell lines and drug sensitivity data of various anticancer drugs against these cell lines. This information is very useful to understand the relationships between drug sensitivity and genomics features of cancer cell lines. In this direction, a few attempts have been made in the past to develop *in silico* models to predict response of cancer cell lines to anticancer drugs. Papillon-cavanagh *et al*., have used two large pharmacogenomics datasets derived from CGP, and CCLE, to build and validate genomic predictors of drug response^8^. In this study, authors have compared five different approaches for building predictors of increasing complexity. In another study, Menden *et al*., have developed machine learning models to predict the response of cancer cell lines to drug treatment^9^. They have used comprehensive drug screening datasets from Genomics of Drug Sensitivity in Cancer’’ (GDSC) project. Neural network models were trained using both the genomic features of the cell lines and the chemical properties of the considered drugs as input features. Similarly Aksoy *et al*. have focused only on homozygous deletions from chemogenomics data of TCGA and CCLE, which may lead to therapeutic vulnerabilities specific to cancer cell as compared to normal cell^10^. Furthermore, recently Berlow *et al*. utilized the CCLE data for predictive modeling of tumor sensitivity of anticancer drugs based on integrated functional and genomic characterizations^11^. In a related study, Geeleher *et al*. fitted models for whole genome expression for three drugs (docetaxel, cisplatin and bortezomib), using large panel of cell lines from CCLE database^12^. Though much attention has been paid in the past to develop various genomic predictors of response of cell lines against anticancer drugs, none of the method is available publicly in the form of web service or software.

In the present study, we used CCLE dataset to analyze different tissues of origin-specific factors as well as genomic factors contributing to drug resistance. We also developed *in silico* models using various techniques for all 24 anticancer drugs (Table 1). These models will be helpful in prioritizing anticancer drugs against a specific cell line from their genomic features. We believe that our models will be useful for researchers working in the field of cancer biology as well as complement the existing methods.

## Results

### Promiscuous vs. Tissue and Cell line specific Drugs

**Figure 1:**
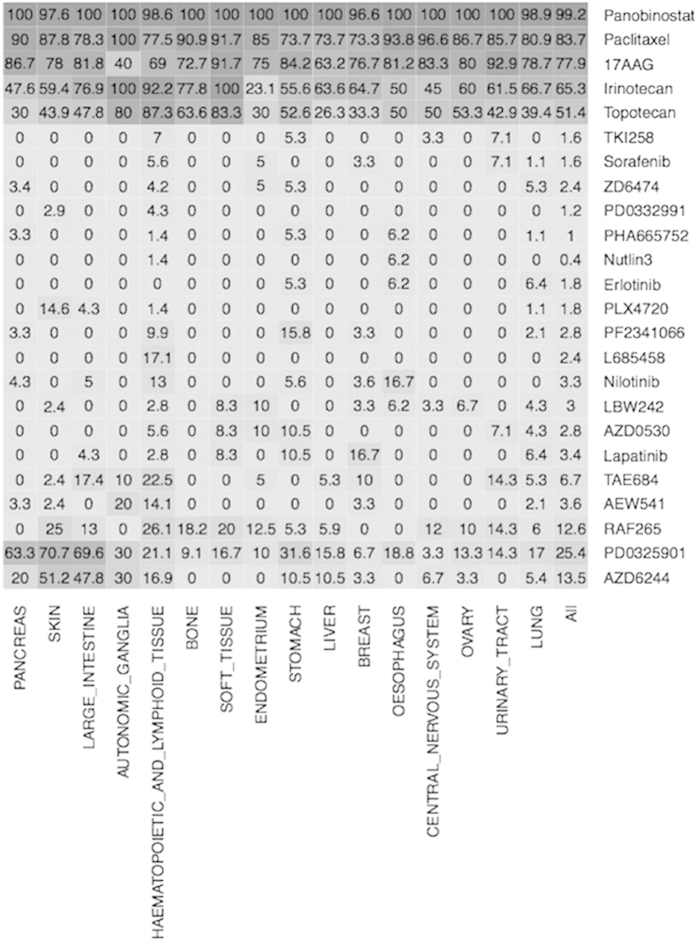
Illustration of tissue-specific response of 24 anticancer drugs, where right column contains names of drugs and bottom row has names of tissues. Each cell shows percent of sensitive cell lines of a tissue for corresponding drug.

We assigned a cell line sensitive to a drug if its growth inhibition IC_50_ value is less than 0.5 μM, otherwise we assigned it resistant. We computed percent of cell lines resistant to each drug as well as percent of cell lines resistant in a tissue (see Supplementary Table S1). It was observed that drug Panobinostat is highly promiscuous anticancer drug, effective against more than 99% cell lines (Fig. 1). Targets of this drug are histone deacetylases (HDAC) or lysine deacetylases (KDAC), enzymes that remove acetyl groups. Another drug Paclitaxel is sensitive against 83% cell lines, as well as it is effective against 100% of the cell lines belonging to Autonomic Ganglia tissue. As shown in Table S1, out of 24 drugs only five drugs (17AAG, Irinotecan, Paclitaxel, Panobinostat, Topotecan) are sensitive against more than 50% of the cell lines assayed. Most of the kinase drugs are only sensitive against limited number of cell lines, whereas most of cytotoxic drugs are sensitive against large number of cell lines. Furthermore, it was observed that certain drugs are tissue specific; for example drug Topotecan is sensitivity against 33% of the breast cell lines (66.7% resistant) where as it is sensitive against 87% cell lines of hematopoietic and lymphoid tissue. Similarly drug Irinotecan is sensitive against 100% cell lines belonging to Autonomic-Ganglia and soft-tissue. There are drugs that are sensitive only against few cell lines like Nutlin3 (effective against less than 1% cell lines) and resistant against most of the cell lines.

### Genomic factors responsible for drug resistance

Each cell line has its own genomic characteristics; these genomic features might be contributing towards drugs resistant. In this study, we investigated role of mutations/variation in genes and gene expression in drug resistant and sensitive cell lines. We identified top five genes corresponding to each drug that exhibit highest difference in genomic features between drug resistant and sensitive cell lines (Supplementary Table S2). Similarly, we also identified genes involved in important activities (e.g., drug membrane transport activity, growth arrest, epigenetic factors, DNA damage, tyrosine protein kinases and tumor suppressors) for each drug (Supplementary Tables S3–S9).

### Variations and mutations

In order to examine the involvement of variation and mutation in drug resistance, we calculated the frequencies of mutant cell lines in both resistant and sensitive group of cell lines (see Methods section) for each gene (Supplementary dataset). In other words, higher the difference of frequencies of mutation between resistant and sensitive cell lines, greater will be the chances of contribution of this gene-mutation combination, in drug resistance. For example, gene *PDE4DIP* (Phosphodiesterase 4D anchoring protein) shows highest difference, it has 38.6% higher frequency of mutation in drug resistant (PF2341066) cell lines as compare to sensitive cell lines (Table 2, Supplementary dataset). It is interesting to note that *PDE4DIP* mutated in 241 cell lines and most of mutant cell lines around 99% were resistant for anticancer drug PF2341066.

While focusing on different biologically relevant functions, we found that DNA damage related proteins like MSH3 and UBR5 genes have >= 12% higher frequency of mutation in TAE684 resistant cell lines as compare to sensitive cell lines (Supplementary Table S7). Similarly, tumor suppressors like TP53 have 19% higher frequency mutation for each of AZD6244 and PD0325901 resistant cell lines (Supplementary Table S9). Among epigenomic factors, SMARCA4 mutations are 11.5% higher in PF2341066 resistant cell lines as compare to sensitive cell lines (Supplementary dataset). PF2341066 resistance may also be brought about by DNA damage related proteins like ATR, FMN2 and UBR5 genes, showing 13%, 14% and 12% higher frequency of mutation in resistant cell lines (Supplementary dataset).

Similar to mutations, we also looked at the presence of variations. We found *PRKCB* (Protein Kinase C, Beta) has 35.7% higher frequency of variation in AZD0530 resistant cell lines as compared to sensitive cell lines (Supplementary Table S2B & Supplementary dataset). Similarly, we found that genes like CYP1A2 (Cytochrome P450, Family 1, Subfamily A, Polypeptide 2) among drug metabolism genes and *SLC22A3* (Solute Carrier Family 22) in drug transmembrane transport activity genes showing higher frequency of variations in TAE684 (18%) and Paclitaxel (13%) resistant cell lines as compared to sensitive cell lines, respectively (Supplementary Table S3 and S4). In contrast, epigenomic factors like *SMARCB1* (SWI/SNF Related, Matrix Associated, Actin Dependent Regulator of Chromatin, Subfamily B; relieves repressive chromatin structures) and *KDM6A* (Lysine K-Specific Demethylase 6A; catalyzes the demethylation of tri/dimethylated histone H3) show as much as 16% more variations in RAF265 and AZD6244 sensitive cell lines, respectively (Supplementary Table S6). Among DNA damage related proteins, *NUAK1* (NUAK family, SNF1-like kinase, 1) and *PLK3* (polo-like kinase 3) were found to be harboring more variations (21% and 18% respectively) in TAE684 resistant cell lines than sensitive ones (Supplementary Table S7).

### Gene Expression

Since the expression of a gene may be associated with the drug resistance, we calculated the average expression of resistant and sensitive cell lines. The difference of two averages shows the relation between expression of that gene and probable drug resistance caused. For example, C3orf14 shows higher average expression (4.5 fold) in AZD0530 resistant cell lines as compare to sensitive (Supplementary Table S2C). Similarly, drug transmembrane transport proteins like ATP8B1 (transport phosphatidylserine and phosphatidylethanolamine across membrane) have high average expression in PD0325901 resistant cell lines as compared to sensitive cell lines, which means its expression may lead to PD0325901 resistance (Supplementary Table S4). In contrast, higher average expression of *TSPAN1* (Tetraspanin 1; involved in cell development, activation, growth and motility) may lead to Topotecan sensitivity (Supplementary Table S4). Moreover, while DNA damage related genes like *CCND1* (Cyclin D1) and *CDC14B* (Cell Division Cycle 14B) have higher average expression in PD0325901 resistant cell lines. *EYA1* (Eyes Absent Homolog 1) was also found to have higher average expression in PD0325901 sensitive cell lines (Supplementary Table S7). Among tyrosine kinases, *ERBB2* and *ERBB3* show high average expression in Topotecan sensitive cell lines (Supplementary Table S8). The tumor suppressors genes such as *EFNA1* (Ephrin-A1), *ERRFI1* (ERBB Receptor Feedback Inhibitor 1), *LATS2* (Large Tumor Suppressor Kinase 2) and *PLK2* (Polo-Like Kinase 2) also show higher average expression in PD0325901 resistant cell lines and thus may be contributing to PD0325901 resistance (Supplementary Table S9).

### Resistance in biologically related group of drugs

In order to understand genomic factors responsible for drug resistance, we group drugs based on biological mechanisms. All the 24 drugs can be broadly divided in three categories; (a) Kinase inhibitors, (b) Cytotoxic drugs and (c) other targeted therapies. These kinase inhibitors can be further divided based on inhibition of a kinase like EGFR inhibitors, c-MET inhibitors, ALK inhibitors, Raf kinase B Inhibitor, MEK1 and MEK2 Inhibitors and Multi-kinase inhibitors. The group of cytotoxic drugs includes inhibition of DNA Topoisomerase-I and Beta tubulin. Other targeted therapies involved in inhibition of MDM2, HSP90, Gamma Secreatase and HDAC. We analyzed mutation/variation of genes in both drug resistant and sensitive cell lines (Supplementary Tables S11 and S12). It was observed that PDE4DIP has 35% higher frequency of mutation in cMET inhibitors’ resistant cell lines as compare to sensitive cell lines. On the other hand, HSPA4 has 49% higher mutation frequency in HDAC inhibitor resistant cell lines. Similarly, MDM2 inhibitors have 46% higher frequency of mutation in sensitive cell lines as compare to resistant cell lines. In case of ABL inhibitors, ADCK1 gene variation is 40% higher in sensitive cell lines.

Similarly, we examined the expression and CNV of different class of inhibitors and identified genes (top 10 and bottom 10 genes) that exhibit maximum correlation between expression/CNV and IC50 (Supplementary Table S13). Here we found certain genes showing good correlation with particular group of drugs, for example, Kinase inhibitors like CDK4/6 inhibitors and ALK inhibitors have correlation of 0.46 and 0.26 with CTTN, which is also known as Src substrate cortactin. Similarly, ETV4 and DUSP6 expression is in negative correlation of 0.4 with MEK1/2 inhibitors’ resistance.

### Prediction Models

In order to develop models for prediction drug prioritization, we developed support vector machine (SVM) based models. In this study, we implemented SVM based regression models using popular software package SVM^*light*^ (http://svmlight.joachims.org/).

### Mutation based models

Since the machine learning approaches required fixed length data, therefore, we took the mutation in a gene in the form of binary where mutated and wild type gene was presented as 1 and 0 respectively. Since the number of features or the vector size for mutational input was 1650, we used cfsSubsetEval algorithm of WEKA package to reduce the number of features^13^. The feature selection with this method reduced the number of genes up to an average of 43 per drug. Still the total number of genes required for modeling all 24 drugs was 388 (Supplementary Table S10). Therefore, we additionally adopted the strategy of F-stepping^14^ on the features obtained from cfsSubsetEval algorithm. Here we looked at the performance of each model by removing every feature one by one as described in Methods section. This process further reduced the number of genes to an average of 20, which are required for modeling of 24 drugs (Supplementary Table S10). After feature selection, the total number of unique genes required for model development for all drugs are 268. SVM based models were developed using selected features and got Pearson Correlation Coefficient (PCC) from 0.24 (Nutlin3) to 0.58 (Irinotecan) with average correlation 0.43 for all drugs. The performances of models after cfsSubsetEval-based feature selection are given in the Supplementary Table S10.

### Models based on gene variation

Similar to the mutational feature, we have taken general variations in a gene as binary input for developing models. The feature selection methods cfsSubsetEval and F-stepping reduced the average number of required genes per drug up to 54 and 25 respectively. After F-stepping filtering on variation data, model development for 24 drugs required a total of 425 unique genes (Supplementary Table S10). As per our hypothesis, the variations present in cancer, which may and may not be mutations, can be used as features to correlate with the drug sensitivity. The average performance of variation-based models in terms of PCC was 0.52 (Table 3), which is better than correlation achieved by mutation based model. In addition we achieved correlation more than 0.60 for certain drugs like Irinotecan, L685458, PD0332991.

### Gene expression based models

SVM-based models developed on gene expression as input features performed very well for all the 24 drugs. The initial feature selection with cfsSubsetEval algorithm reduced the average number of required genes for model development up to 66. The total number of unique genes required for 24 drugs, were 1296 in number (Supplementary Table S10). We further reduced the average required genes up to 28 with total 619 unique genes using feature selection technique F-stepping (Supplementary Table S10). Subsequently, the models were developed with these selected numbers of genes. The mean PCC for models of all the drugs was 0.73 (Table 3), which was significantly higher than the correlation achieved using models based on models and variation. Our expression based models got high correlations more than 0.8 for certain drugs like AZD6244, Irinotecan, Nilotinib, PD0332991. The performance of models without using F-stepping is also provided in the Supplementary Table S10.

### Models using copy number variations

Since the copy number variation (CNV) deals with alteration of genome with large regions resulting in deletion or duplication of many genes, we have also taken CNV as one of the features for predictive modeling. The average number of genes required for all 24 drugs were 45 and 21, before and after F-stepping respectively. After F-stepping, the final models required a total of 877 unique genes (Supplementary Table S10). With an average Pearson correlation of 0.55 for all 24 drugs, CNV as a feature performed better than mutation and variation but less than expression. It is interesting to note that we got high correlation 0.71 and 0.68 for drug Nilotinib and PLX4720 respectively.

### Models based on hybrid features

In order to improve performance of our predictive models, we used hybrid features. In case of hybrid features, we combined two or more than two types of genomic features. First, we generated hybrid features by combining genomic features based on mutation, expression and CNV. These hybrid features were reduced using feature selection techniques cfsSubsetEval and F-stepping and achieved average number of genes up to 80 and 34 respectively (Supplementary Table S10). These reduced hybrid features were used for developing prediction model and achieved average correlation of 0.78, which is better than models developed using different sets of features separately. Similarly, we also developed a number of models using combination of genomic features; none of the models got average correlation more than 0.78. In addition our models were highly successful to certain drugs like LBW242, PLX4720 where correlation was 0.9.

#### Significant mutational features

In order to further minimize the number of genes for modeling, we looked at the drugs, which showed significant difference of IC_50_ between mutated and normal cell lines for a given gene. For this, we minimized the number of drugs to as few as 7 drugs, which led us to 30 genes with significant difference (p-value < 0.05) between IC_50_ of mutated and normal cell lines. Further, we performed feature selection on significant genes, by F-stepping. Our SVM^*light*^ based models achieved a maximum correlation of 0.22 using selected features (Supplementary Table S10). We have included these models in our web server for mutation-based module, which provided drug prioritization prediction with minimum number of genes.

#### Significant variation features

Similar to significant genes in mutation as given above, we also looked at significant genes keeping variations in mind. We got 32 significant genes, which cover at least 7 drugs. We incorporated the variation profile of these genes as machine learning input and our best model achieved correlation of 0.22. The drug wise performances are mentioned in Supplementary Table S10.

#### Correlated expressional features

Similar to the significant genes in mutation, we also identified genes whose expression shows high correlated with growth inhibition IC_50_. We selected top 50 such genes, which are highly correlated for all drugs. Our SVM model, based on these 50 genes, attained average correlation of 0.46 for all 24 drugs (Supplementary Table S10).

### Drug targets *vs* selected features

We computed correlation between drug targets and genes selected for developing prediction models to understand relationship between targets and selected features (Supplementary Table S14 to S17). It was observed that number of drug targets have good correlation with genomic features. As shown in Table S14, number of genes selected for developing mutation-based models also includes drug targets (e.g., EGFR, FLT3). In addition, number of drug targets shows high correlation with genes selected for developing prediction model; for example CDK6 shows high correlation 0.41 with gene PDPK1 selected for developing mutation based model for drug PD033299. In case of expression-based models, ALK (target of TKI258) and model gene IL26 have a correlation of 0.58 in expression. In CNV-based models also the target of Nilotinib (ABL1) is in good correlation (0.9) with model genes like QRFP and NUP214 (Supplementary table S16 & S17). In variation-based models, genes selected for developing model for drugs (e.g., 17AAG, PD0325901) also include their drug targets (e.g., HSP90AA1, MAP2K1). Expression-based model for Nutlin includes the drug target MDM2 in the selected model genes. Furthermore, among CNV-based models, drug targets ERBB2 (Lapatinib) and MET (PF2341066) are part of genes selected for developing prediction models.

### Comparison with existing study

In the past, several large-scale pharmacogenomics studies have been done with human cancer cell lines, for example, Cancer Cell Line Encyclopedia (CCLE), NCI-60 and Cancer Genome Project. As shown in Table 3, most of our models perform better than CCLE models developed in previous study. CCLE models got maximum correlation 0.43 where as our best model achieved average correlation up to 0.78.

### Implementation of web server

In order to serve the scientific community working on cancer biology, we have developed a user-friendly webserver for predicting growth inhibition of anticancer drugs against a cancer cell using its genomic features. In addition, various useful tools are also integrated which assist users in identifying most effective drugs. Brief description of major modules is given follows:

### Prioritization

This module is built on the basis of our machine learning models developed based on mutations, variations, expressions, CNVs and their hybrid as input features. User may submit related genomic information or raw NGS data (VCF/ANNOVAR input file)^15^ and subsequently server will predict the effectiveness of drugs based on models developed in this study.

### Drug calculator

This module has been built in order to find out the contribution of each gene in drug prioritization. This module is based on probabilistic approach. First, we have divided the cell lines into resistant (IC_50_ > 0.5 μM) and sensitive (IC_50_ <= 0.5 μM) cell lines for each of 24 anticancer cells. Then, we developed modules based on various genomic features as shown below.

#### Mutation and variation-based modules

After classifying the cell lines into resistant and sensitive cell lines, then we computed probability (frequency) of finding the mutations/variations in a particular gene in both resistant and mutant cell lines. User has to enter the HUGO symbol of mutated/variant gene(s) and this module will return the probability values of finding mutations/variations in both resistant and sensitive cell lines.

#### Expression and CNV-based modules

In these modules, we have calculated the average expression/CNV of a particular gene in resistant and sensitive cell lines together with their differences (DIFF values) for 24 anticancer drugs. By looking at these values user can identify, which drug will be more effective for cell line having high/low expression/CNV of the query gene.

### Signature module

The purpose of this module is to identify important genes corresponding to each drug. In case of mutation, we computed average IC_50_ of a drug for each gene in mutant & wild cell lines to understand effect of mutation on drug sensitivity or resistance. In case of expression, we computed average expression of each gene for resistant & sensitive cell lines for a given drug.

#### Mutation/variation based

In these modules, server displays each gene used in this study and average growth inhibition of selected drug in cell lines contains mutated and wild form of a gene. This module also displays difference in growth inhibition with significance (p-value) in difference. These modules provide comprehensive information about a selected drug in terms of effect of mutation or variation of a gene on its growth inhibition.

### Expression and CNV Based

The aim of this tool is to find out the difference between average expression/CNV of a particular genes in resistant as well as sensitive cell lines. It allows the user to select a desired drug; then these modules display a list of all genes with their average expression/CNV in resistant and sensitive cell lines. These modules are very useful in understanding effect of expression/CNV of a gene on growth inhibition of a drug.

## Discussion

In the era of high-throughput technologies, several efforts have been made to unveil the complex relationship between the genetic features and the drug response. In this direction, Broad Institute carried out a magnificent study, where they generated huge amount of pharmacogenomics data. This study paved the path for researchers to understand relation between genomic features of a cell and growth inhibition abilities of a drug on this cell. In this study, we also used this data for identification important genomic characteristics responsible of drug resistance. In addition, we also developed prediction models for predicting growth inhibition of each anticancer drug against a cell lines from genomic features of cancer cell line. As shown in Results section, certain drugs are highly promiscuous as they kill almost all types of cell lines. It seems that their growth inhibition ability was hardly affected by alteration of genomic profile of cells. In contrast, drugs like Erlotinib, LBW242, Nutlin3, PD0332991 are effective against very limited types of cell lines; resistant to most of cell lines.

We examined the tissue-specific response of different drugs and observed that certain drugs are tissue specific where they inhibit almost all cell lines belongs to specific tissue. As shown in Table S1, drugs showed different level of response to cell lines belonging to different tissues. It may be due to fact that cell lines belonging to different tissues have different genomic features. We also examined the effect of mutations and variations in genes involved in important processes or pathways. It was Our analysis shows that tumor suppressor like TP53 have 19% higher frequency mutation for each of AZD6244 and PD0325901 resistant cell lines. Since both are MEK inhibitors, there may be some common factor related to TP53 mutation, which might be contributing to resistance to both the drugs. Similarly, it was observed that epigenetic enzymatic SMARCA4 plays important role in drug resistance. Earlier studies also have shown that mutation in *SMARCA4* gene have been associated with lung cancer, medullablastoma and pancreatic cancers etc.^16^,^17^,^18^.

Similarly, the difference in the average gene expression of resistant and sensitive cell lines may be associated with drug resistance; for example ATP8B1 belongs to transport proteins, have a higher average expression in PD0325901 resistant cell lines as compared to sensitive cell lines. ATP8B1 (ATPase class I type 8B member 1) is an ATP-dependent aminophospholipid transporter, which has a major role in the transport of endogenous chemicals across the biological membranes^19^,^20^. ATP8B1 had a higher expression in PD0325901 resistant cell lines, which may be responsible for the efflux of the drug in cancerous cell lines and thus resistance. CCND1 is a cell cycle regulatory protein that has a role in G1/S cell cycle checkpoint that monitors for unrepaired DNA damage. Earlier *in vitro* studies have shown a positive correlation between increased expression of CCND1 and resistance to Cisplatin in head, neck and colon cancer cells^21^,^22^. Inhibition of CCND1 may increase the sensitivity to drugs in case of pancreatic cells. Similarly, our studies show that there was higher average expression of DNA damage related proteins like CCND1 and CDC14B in PD0325901 resistant cell lines thus increased expression may lead to increased PD0325901 resistance.

Our study with grouped drugs based on underlying mechanism, lead us to different genomic factors associated with drug resistance. As shown in the results that PDE4DIP mutation is more frequent in cMET inhibitors, which means mutations in PDE4DIP may lead to resistance towards cMET inhibitors. Similarly, DUSP6 and ETV4 expression showed a negative correlation with MEK1/2 inhibitors and thus an increased expression of these genes may lead to MEK1/2 inhibitors’ resistance. Previous reports of genome wide screening for biomarkers for MEK1/2 sensitivity also reported DUSP6 and ETV4 as markers for MEK1/2 sensitivity^23^,^24^.

We made an attempt to develop prediction models based genomic profile of cell lines. After various feature selection techniques, the machine learning-based models for each drugs required very few number of genes for prediction (average number of genes per drug model in mutation = 20, variation = 25, expression = 28, CNV = 21). Among the selected genes for model, some of the genes were targets for that particular drug. One of the major problems in developing prediction model is identification of features or biomarkers. Unfortunately, there is no clear-cut biomarker, which can discriminate drug resistant and sensitive cell lines. If we develop models using large number of features then it has limited use in real life. Ideally models should be developed using very limited features. In this study we have used cfsSubsetEval algorithm and F-stepping technique^14^ for selecting minimum number of features without compromising the performance of models (Table 3). In addition to the machine learning-based models, we also developed propensity-based models using genomic features in order to overcome limitation of SVM based models that require fixed length patterns. These models are suitable even if user has information about limited set of genes. The propensity-based models provide contribution of every single gene along with sum of all the genes. This will help user to look at better drug with fewer genes. Such probability-based prediction enables us to examine genes, which may contribute to drug resistance or sensitivity by alteration in genomic features like mutation, variation, expression and CNV.

## Methods

### Dataset

In this study, we used CCLE^7^ cancer drug screening dataset, which contained compound screening data performed on large panels of molecularly characterized cancer cell lines. The sequencing data includes hybrid capture sequencing of 1667 genes in 448 cell lines and provides two different type of information- (i) Mutations: without common SNPs and neutral variants and (ii) Variations: all variations, which were present in the gene of particular cell line. The expression data includes RMA-normalized mRNA expression data of 17627 genes in 488 cell lines (http://www.ebi.ac.uk/arrayexpress/experiments/E-GEOD-36139/). Similarly normalized CNV data for 21217 genes of 418 cell lines was obtained from Broad institute’s CCLE portal (http://www.broadinstitute.org/ccle/home). The drug sensitivity information for 504 cell lines was captured from CCLE browse data section. The data includes IC_50_ (μM) for 24 anticancer drugs on 504 cell lines. Out of these 24 anticancer drugs, 9 drugs are launched, 2 are in phase III trial, 3 are in phase II trial and 7 are in preclinical trial.

### Analysis for drug resistance and sensitive cell lines

In order to understand the role of gene mutation in drug resistant cell lines, we computed biasness of mutated genes in sensitive and resistant cell line. In order to compute biasness, we computed difference in fraction of mutant in resistant and sensitive cell lines. Firstly, we computed total number of resistant and sensitive cell lines of each drug. Secondly, we computed fraction of cell lines having mutated gene for a given gene. Following equation has been used for computing difference in fraction


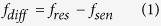


where, *f*_*res*_ is the fraction of number of resistance cell lines having mutated genes with total number of resistant cell lines. Similarly, *f*_*sen*_ is the fraction of number of sensitive cell lines having mutated genes with total sensitive cell lines.

Similar approach has been used in case of variation for assessing role of variation in drug resistant. We computed difference in fraction of variant gene in resistant and sensitive cell lines using equation 1. In case of gene expression and copy number variation, we computed correlation coefficient between IC_50_ and gene expression or copy number variation.

### Features for developing models

#### Mutation

It is well known that mutations play important role in cancer progression and also affects the sensitivity of drug molecules. Thus, we used mutation as an input features for developing prediction model using machine-learning approaches. The mutations in a gene were given in IUPAC nomenclature standard, as a mutation annotation file (MAF). Since the IUPAC mutation annotations, cannot be used in machine learning as input feature *per se*, we had to convert those into binary format. The mutated gene and normal gene (not mutated) were represented as ‘1’ and ‘0’ respectively. The binary (1 or 0) status for every gene was used as machine learning input. For example mutated gene G1 and normal gene G2 were presented as ‘1’ and ‘0’ respectively.

#### Variation

As we know that the rate of variation is high in cancerous cells and among these raw variations, some rare deleterious alterations are called mutations. We, for the first time, have considered general variations as players in the drug sensitivity. The presence and absence of such variations was taken as binary input and represented as ‘1’ and ‘0’ respectively. For example, gene G1 having variation was presented as ‘1’ and gene G2 not having variations was presented as ‘0’.

#### Gene expression

The expression profile of 488 cancer cell lines was obtained from CCLE database. The authors of CCLE database obtained the mRNA expression data using Affymetrix Human Genome U133 Plus 2.0 arrays as per the manufacturer’s instructions. The background correction was accomplished by RMA (Robust Multichip Average) and quantile normalization. The provided expression values were given in log2 of expression value. In this study we used these normalized value of expression as input vector for developing prediction model.

#### Copy Number Variation (CNV)

Similar to the expression data, CNV information for 418 cell lines was obtained from CCLE database. According to the authors, the raw Affymetrix CEL files were converted to a single value for each probe set representing a SNP allele or a copy number probe. CNV values are given in log2 of ratio of copy numbers of cancer vs. normal gene, where positive and negative values denote increase and deletion in copy numbers of the gene respectively. These values were used as input features for developing SVM based prediction model.

### Feature selection

#### Feature selection using CfsSubsetEval algorithm of WEKA

This algorithm calculates the significance of a subset of attributes by considering the individual predictive ability of each feature along with the degree of redundancy between them. Finally, subsets of features, which are having low inter-correlation and are high correlation with the IC_50_, are selected. We applied this algorithm through WEKA [Version 3.6.6]. We applied cfsSubsetEval algorithm on whole mutation, variation, expression, CNV and hybrid data.

#### Feature selection by F-stepping method

This method has been applied in the past for feature selection^14^. In this method we leave one feature out, and then we develop and evaluate the performance of model without a given feature. If the performance remains equal or increases, we remove the feature else if by removing the feature performance gets down we keep it and move to next cycle with other feature. This way we got those features that are important for developing models and removed useless or least important features.

#### Feature selection by correlation (expression vs. IC_50_)

To reduce the number of features we computed the correlation between IC_50_ with expression of genes. We selected top 50 most correlated genes irrespective of positive or negative relation. We used expression of these genes as input feature for machine learning methods.

#### Feature selection by Significance (Mutated vs. Normal gene)

In case of mutation of genes, where the mutations were either present (denoted as 1) or mutations were absent (denoted as 0), we selected those genes which were showing significant difference (p-value < 0.05) of IC_50_ between mutated cell lines and not-mutated cell lines for a particular gene.

#### Genomic features from Next Generation Sequencing (NGS)

In addition to the list of mutated/variant genes as described above, we have provided the option of submitting raw Variant Calling Format (VCF) file, which is one of the most commonly used format for storing mutations/variations after NGS. Since the size of VCF files may be problematic in uploading because low speed internet network, we have also provided option for submitting ANNOVAR input file. VCF file can be easily converted to ANNOVAR input file by given link of downloadable PERL script. The smaller sized ANNOVAR file can easily be uploaded via internet. The ANNOVAR input or VCF submission files are processed with ANNOVAR package (Version Revision: 527). The steps include the processing of ANNOVAR input/VCF file, extraction of annotated variation in different genes, filtering of SNPs (present in dbSNP^25^) to get mutations present in different genes, identification of mutated signature genes and machine learning based prediction with signature genes (as described above). The variations based prediction use the extracted annotations without SNP filtering.

## Conclusion

The predictive modeling for anticancer drug sensitivity has been a very meticulously studied area in cancer biology. In spite of several large-scale studies, we still do not have any general rules/guidelines in public, for which anticancer drug should be preferred over other drugs. There are number other issues which have not been addressed in this study that includes toxicity of drugs as all drugs are not suitable to all patients. In this study, models have been developed using different genomic features; each type of models have their own strength and weakness. First we developed mutation-based models, unfortunately the performance of these models were too poor. Ideally mutation in target gene of a drug should effect its sensitivity but there are number of drugs whose target gene is heavily mutated but no effect on sensitivity. In contrast there are number of drug resistant cell lines despite there is no mutation in their drug targets. The best mutation model achieved maximum correlation 0.68 between actual and predicted IC_50_ value for drug PLX4720. It was observed (Table 2) that there are certain genes that are heavily mutated in resistant cell lines for number of drugs. The mutation in gene TP53, KRAS and MAP3K1 significantly affect sensitivity of ten drugs. We have not observed any biasness in models towards kinase or cytotoxic drugs.

In this study, first time we used variation for developing models, we believed that variation may also affect drug sensitivity without affecting the function of gene. As expected variation based models perform better than mutation-based models. Thus it is important to use variation-based models instead of mutation-based models. In addition identification of variation is easy in comparison to identification of mutations. As shown in Table 3, expression based models over perform other models and achieved average correlation around 0.73. One of the advantages of these models is that measuring expression of genes is easy in comparison to identification of mutations/variations. The expression of genes has dynamic nature it change with time and conditions. We also explore another genomic feature CNV that is highly correlated with gene expression. Though performance of CNV-based models was poorer than expression-based models but better than other models. One of the major advantages of CNV based model is that this genomic feature has more genetic basis than environmental effect.

As shown above each type of model has their own merits and demerits, thus we implement all models in our web server. This will allow users to select best model for predicting sensitivity of drugs. In this study, for the first time, we also made an attempt to develop a webserver-based anticancer drug prioritization tool, which is a initial step towards personalized drug therapy for cancer. Scientific community can used our webserver at least for studying drugs on cancer cell lines (http://crdd.osdd.net/raghava/cancerdp/).

## Additional Information

**How to cite this article**: Gupta, S. *et al*. Prioritization of anticancer drugs against a cancer using genomic features of cancer cells: A step towards personalized medicine. *Sci. Rep.* **6**, 23857; doi: 10.1038/srep23857 (2016).

## Supplementary Material

Supplementary Information

## Figures and Tables

**Table 1:** List of 24 anticancer drugs used for the development of *in silico* models along with their clinical status.

S. NO.	Drug (Generic Name)	Target	Type of Inhibitor	Clinical Status
1	AEW541	IGF-1R	Kinase	Preclinical
2	AZD0530	Src, Abl/Bcr-Abl, EGFR	Kinase	Phase II
3	AZD6244	MEK	Kinase	Phase II
4	Erlotinib	EGFR	Kinase	Launched
5	Lapatinib	EGFR, HER2	Kinase	Launched
6	Nilotinib	Abl/Bcr-Abl	Kinase	Launched
7	PD-0325901	MEK	Kinase	Discontinued
8	PD-0332991	CDK4/6	Kinase	Phase II
9	PF-2341066	c-MET, ALK	Kinase	Launched
10	PHA-665752	c-MET	Kinase	Preclinical
11	PLX4720	RAF	Kinase	Preclinical
12	RAF265	Raf kinase B, KDR	Kinase	Phase I
13	Sorafenib	Flt3, C-KIT, PDGFRbeta, RET, Raf kinase B, Raf kinase C, VEGFR-1, KDR, FLT4	Kinase	Launched
14	TAE684	ALK	Kinase	Preclinical
15	TKI258	EGFR, FGFR1, PDGFRbeta, VEGFR-1, KDR	Kinase	Phase III
16	Vandetanib	Abl, EGFR, Flt3, C-KIT, RET, VEGFR-1, KDR, FLT4	Kinase	Launched
17	Irinotecan	Topoisomerase I	Cytotoxic	Launched
18	Paclitaxel	Beta-tubulin	Cytotoxic	Launched
19	Topotecan	Topoisomerase I	Cytotoxic	Launched
20	17-AAG	HSP90	Other	Phase III
21	L-685458	Gamma Secretase	Other	Preclinical
22	LBW242	IAP	Other	Preclinical
23	Nutlin-3	MDM2	Other	Preclinical
24	Panobinostat	HDAC	Other	Registered

**Table 2:** Gene showed most biased mutation (fraction of mutant cell lines is more in resistant than in sensitive cell lines) for each anticancer drug.

Drug	Gene	Total	Resistant Cell Lines			Sensitive Cell Lines			Fraction Difference
			Mutant	Total	Fraction	Mutant	Total	Fraction	
17AAG	SPEN	447	21	97	0.216	38	350	0.109	0.107
AEW541	MLL3	447	128	430	0.298	1	17	0.059	0.239
AZD0530	MAP3K1	448	339	434	0.781	8	14	0.571	0.21
AZD6244	TP53	447	249	382	0.652	30	65	0.462	0.19
Erlotinib	TTN	447	321	438	0.733	3	9	0.333	0.4
Irinotecan	KRAS	279	30	97	0.309	33	182	0.181	0.128
L685458	KRAS	435	90	423	0.213	0	12	0	0.213
LBW242	MLL3	435	125	423	0.296	1	12	0.083	0.213
Lapatinib	MSH3	447	148	434	0.341	2	13	0.154	0.187
Nilotinib	LRP1B	370	109	358	0.304	0	12	0	0.304
Nutlin3	TP53	448	280	446	0.628	0	2	0	0.628
PD0325901	TP53	448	223	330	0.676	57	118	0.483	0.193
PD0332991	MAP3K1	384	295	379	0.778	2	5	0.4	0.378
PF2341066	PDE4DIP	448	241	436	0.553	2	12	0.167	0.386
PHA665752	NCOA3	447	200	444	0.45	0	3	0	0.45
PLX4720	TP53	440	274	431	0.636	1	9	0.111	0.525
Paclitaxel	GPR112	447	54	71	0.761	241	376	0.641	0.12
Panobinostat	HSPA4	444	2	4	0.5	4	440	0.009	0.491
RAF265	KRAS	408	81	355	0.228	4	53	0.075	0.153
Sorafenib	CREB3L2	447	226	439	0.515	1	8	0.125	0.39
TAE684	CSMD3	448	105	416	0.252	2	32	0.062	0.19
TKI258	AAK1	448	241	441	0.546	1	7	0.143	0.403
Topotecan	KRAS	448	60	220	0.273	32	228	0.14	0.133
ZD6474	CREB3L2	440	224	430	0.521	2	10	0.2	0.321

**Table 3:** The performance of SVM models developed using various genomic features that include mutant genes, variant genes, CNV, expression, hybrid.

Drug	Mutation	Variation	Expression	CNV	Hybrid	CCLE*
17AAG	0.42	0.55	0.67	0.54	0.76	0.43
AEW541	0.25	0.54	0.69	0.54	0.75	0.33
AZD0530	0.41	0.45	0.65	0.56	0.71	0.19
AZD6244	0.52	0.51	0.81	0.56	0.82	0.59
Erlotinib	0.48	0.56	0.79	0.62	0.82	0.3
Irinotecan	0.58	0.65	0.84	0.56	0.87	0.68
L685458	0.44	0.63	0.82	0.59	0.89	0.48
LBW242	0.44	0.52	0.72	0.52	0.90	0.46
Lapatinib	0.43	0.57	0.75	0.64	0.79	0.09
Nilotinib	0.58	0.53	0.84	0.71	0.77	0.76
Nutlin3	0.24	0.26	0.52	0.33	0.62	0.1
PD0325901	0.54	0.50	0.82	0.55	0.83	0.6
PD0332991	0.42	0.61	0.84	0.51	0.87	0.62
PF2341066	0.38	0.56	0.75	0.61	0.74	0.62
PHA665752	0.37	0.49	0.60	0.49	0.70	0.49
PLX4720	0.68	0.56	0.79	0.68	0.90	0.38
Paclitaxel	0.34	0.51	0.58	0.48	0.73	0.29
Panobinostat	0.46	0.50	0.78	0.58	0.82	0.58
RAF265	0.48	0.49	0.73	0.53	0.78	0.35
Sorafenib	0.37	0.58	0.78	0.44	0.76	0.28
TAE684	0.38	0.42	0.68	0.52	0.74	0.38
TKI258	0.36	0.43	0.72	0.53	0.76	0.3
Topotecan	0.44	0.55	0.75	0.54	0.80	0.58
ZD6474	0.36	0.48	0.71	0.53	0.74	0.22
**Average**	**0.43**	**0.52**	**0.73**	**0.55**	**0.78**	**0.42**

The performance is given in the form of correlation coefficient between predicted and actual IC_50_.

^*^The performance of models developed in CCLE study.
